# Photovoltaic Module Degradation Detection Using V–P Curve Derivatives and LSTM-Based Classification

**DOI:** 10.3390/s25206475

**Published:** 2025-10-20

**Authors:** Chan-Ho Lee, Sang-Kil Lim, Sung-Jun Park, Beom-Hun Kim

**Affiliations:** 1 Department of Electronic Engineering, Chosun University, Gwangju 61452, Republic of Korea; dlcksgh2948@chosun.kr (C.-H.L.); sklim@chosun.ac.kr (S.-K.L.); 2 Department of Electrical Engineering, Chonnam National University, Gwangju 61186, Republic of Korea; sjpark1@jnu.ac.kr; 3 Department of Information Communication Engineering, Chosun University, Gwangju 61452, Republic of Korea

**Keywords:** photovoltaic systems, PV module degradation, voltage–power curve, degradation detection, derivative, long short-term memory (LSTM), AI-based diagnostic model

## Abstract

Photovoltaic systems are a core component of eco-friendly energy technologies and are now widely utilized across the world for power generation. However, solar modules that are continuously exposed to the external environment experience gradual performance degradation, which results in significant power loss and operational problems. Existing aging diagnostic methods such as current–voltage curve analysis and electroluminescence/photoluminescence testing have limitations in terms of real-time monitoring, quantitative evaluation, and applicability to large-scale power plants. To address these challenges, this study proposes a novel degradation detection method that utilizes the first-order derivative of the voltage–power curve of solar modules to extract key features. This method can estimate the number of degraded solar modules within a string and the degree of degradation, enabling early detection of subtle changes in electrical characteristics. In this study, we developed an AI model based on long short-term memory to classify normal and abnormal states and predict aging status, thereby supporting monitoring and early diagnosis. The model architecture was designed to reflect the characteristics of solar power systems, adopting a relatively shallow network due to the time-series data not being excessively long and the feature changes being clear. This design effectively mitigates the issues of overfitting and gradient vanishing, thereby positively contributing to the stability of model training. The training and validation results of the proposed long short-term memory model were verified through MATLAB simulations, confirming its effectiveness in learning and convergence.

## 1. Introduction

Photovoltaic(PV) systems are a major power generation method that is widely and reliably used around the world for sustainable energy [1]. However, external stressors (UV, temperature/humidity variation, shading) cause gradual PV-module degradation, reducing annual power output by 0.5–1% and yielding losses of tens to hundreds of GWh [2]. This negatively affects plant operators’ profitability and progress toward national renewable-energy targets [3]. In PV systems, solar modules are typically configured in series and parallel. In series- parallel configurations, string-level degradation becomes current-limiting, lowering efficiency and shifting the maximum power point (MPP) downward; this, in turn, increases maintenance costs, impairs operational stability, and reduces overall efficiency [4]. Therefore, it is essential to develop a technology that can accurately and promptly diagnose the degradation of PV modules. Existing solar module diagnostic techniques mainly include current–voltage (I–V)characteristic curve analysis and electroluminescence (EL)/photoluminescence (PL) testing. While recent studies utilizing the entire I–V curve with models like PCA, 1D-CNN, and LSTM have achieved accuracy exceeding 99% [5], on-site I–V measurement requires precise equipment and calibration and can cause power generation losses [6]. EL/PL is useful for visualizing micro-defects, but dark-room/power requirements and the risk of missing early degradation limit large-scale deployment [7]. Recent deep and machine learning methods using power-generation data succeed in anomaly classification and power-output forecasting but offer limited capability to quantify degradation severity [8,9]. In addition, most AI models focus on anomaly detection at the power plant or string level, which limits their ability to accurately analyze the status of individual modules [10,11]. In this study, degradation features are extracted from the first-order derivative of the voltage–power (V–P) curve and then applied to a two-stage, long short-term memory (LSTM)-based classification model. In the first step, normal and abnormal states are classified. In the second step, the number and degree of degraded panels are quantitatively predicted, but only for the data identified as abnormal. By combining V–P curve derivative features with a hierarchical LSTM, precise early diagnosis that overcomes the limits of conventional methods becomes possible. A real-time system based on this approach can minimize generation losses and enhance operational stability by enabling prompt operator responses.

**Figure 1:**
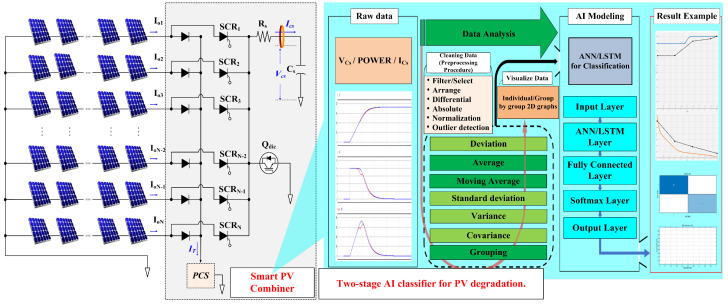
Smart PV combiner and AI-Based PV module degradation classification system.

Figure 1 shows a schematic diagram of the smart PV combiner and AI-based panel-degradation classification system to be developed in this study. Figure 1 is a conceptual diagram of the proposed system. PV-string electrical outputs are collected via an silicon controlled rectifier (SCR)–R–C-based smart PV combiner, preprocessed, and analyzed by a two-stage AI model to diagnose degradation. Through this, it is expected that the status of each module can be effectively managed and power generation efficiency maximized when operating large-scale solar power plants.

The remainder of this paper is organized as follows. Section 2 provides background on PV module degradation; Section 3 describes the SCR–R–C-based V–P detection method and its circuit operation. Section 4 details the AI-based diagnostic model (normal/abnormal grouping and stage classification), Section 5 reports the experimental results, and Section 6 concludes with future directions.

## 2. Theoretical Background of Solar Module Degradation

Solar panels undergo various forms of degradation over time due to external environmental factors such as ultraviolet exposure, temperature and humidity fluctuations, shading, and electrical loads after installation. These mechanisms include mechanical damage manifested as microscopic cell cracks, potential-induced degradation (PID), which triggers abrupt performance drops from voltage differentials, and the hot-spot phenomenon, where localized overheating causes panel deterioration.

### 2.1. Microcrack

**Figure 2:**
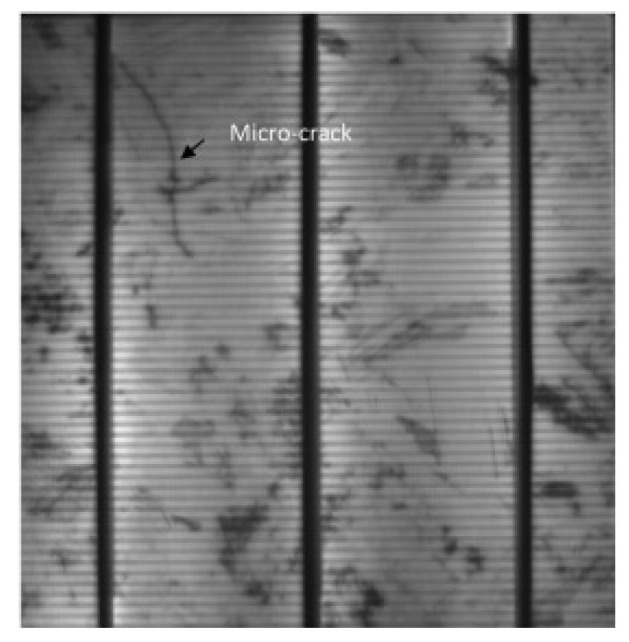
Microcrack in a polycrystalline solar cell.

**Figure 3:**
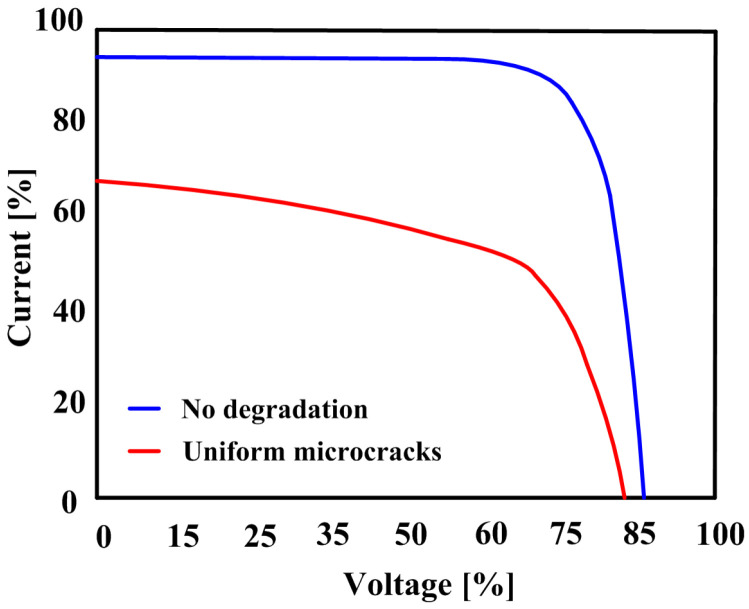
Microcracks degradation effect on I–V curve.

Microcracks in PV cells develop in the silicon wafer due to external impacts, thermal cycling, and mechanical stress. Microcracks, as shown in Figure 2, are difficult to identify with the naked eye but can be detected via EL patterns [12]. By increasing local series resistance and reducing effective conductive paths, they degrade the I–V characteristics, as shown in Figure 3 [13]. Typically, the short-circuit current ($I_{SC}$) decreases by approximately 30%, leading to a corresponding drop in the open-circuit voltage ($V_{OC}$). This reduction in low-voltage current lowers efficiency and increases accumulated power loss at the string level.

### 2.2. PID (Potential Induced Degradation)

Solar PID is a degradation phenomenon that occurs when a potential difference of several hundred to several thousand volts is generated between PV modules and frames, causing leakage current to flow within the cells and at the junctions [14]. During this process, Na+ ions from the glass substrate diffuse toward the silicon cells, or micro-short circuits form between the electrodes, causing a sharp decrease in surface resistance. As a result, the open-circuit voltage ($V_{OC}$) and MPP decrease significantly, and the reduction in fill factor(FF) is particularly noticeable in the I–V curve. FF is the maximum power of the solar cell by the short-circuit current ($I_{SC}$) and $V_{OC}$, and can be expressed by the following Equation (1).

(1) $$FF=\frac{P_{MP}}{V_{OC}*I_{SC}}$$

**Figure 4:**
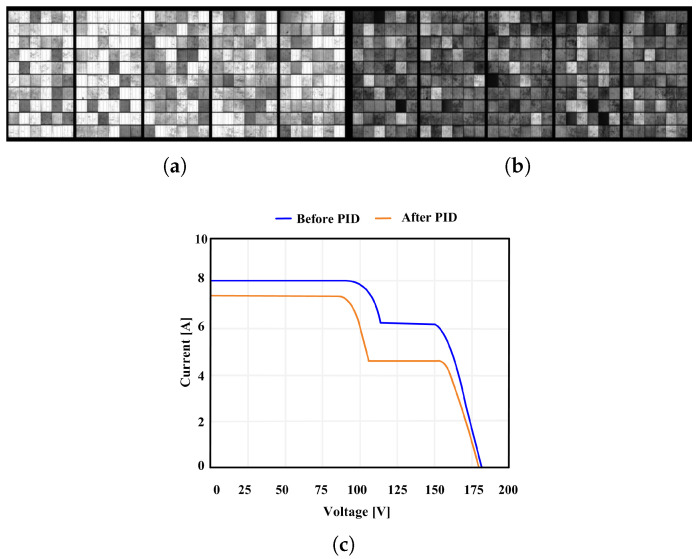
EL images and I–V characteristics of a PV module. (**a**) Before PID; (**b**) after PID; (**c**) I–V curves before and after PID.

Figure 4 shows PID-induced degradation via EL imaging. The luminescence intensity and spatial consistency observed in EL images are closely associated with the condition and performance of photovoltaic cells, where darker regions typically indicate degradation caused by PID. Figure 4a (pre-PID) shows strong, uniform emission, whereas Figure 4b (post-PID) shows darkened areas. The changes in the I–V characteristics caused by PID are shown in Figure 4c, where a decrease in $I_{Cs}$ and MPP, as well as a degradation of FF, are observed due to leakage paths [15]. The images were adapted from the work of Dhimish and Badran [15].

### 2.3. Hot Spot

A hot spot occurs when shading, contamination, or other factors reduce the output of some cells/strings, forcing reverse bias on the remaining cells and causing localized overheating. Because string current must remain constant, underperforming cells conduct in reverse; the reverse bias dissipates power as heat, raising cell temperature [16]. If the hot spot persists, cell damage may occur, potentially leading to a shutdown of power generation. Furthermore, when only part of the string deteriorates rather than the entire cell, the slope of the V–P curve becomes asymmetrical, and this change can be effectively detected using a first-order derivative-based degradation detection technique [17].

In addition, various other degradation mechanisms exist that can reduce the performance of photovoltaic modules. PV modules are subject to Light-Induced Degradation (LID), which causes a rapid decline in performance due to the activation of boron–oxygen complexes upon initial light exposure, and to Light and elevated Temperature-Induced Degradation (LeTID), which results in additional performance loss under prolonged exposure to high temperatures [18]. The stress factors affecting failures in photovoltaic modules and the resulting performance degradation are summarized in Table 1.

### 2.4. The Effect of Solar Module Degradation on Electrical Characteristics (V–P Curve)

**Figure 5:**
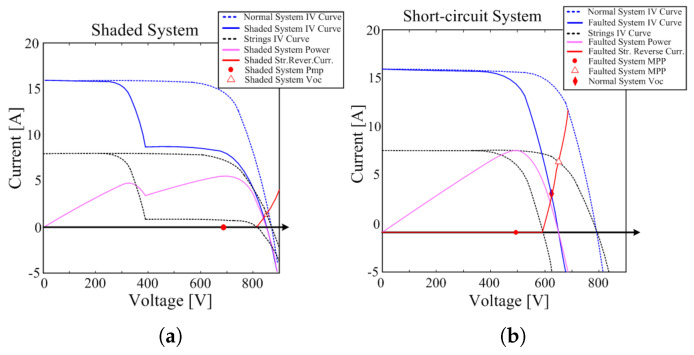
Reverse current in a (**a**) shaded; (**b**) short-circuited string.

As solar modules degrade, various characteristic changes appear in the V–P curve. As the internal series resistance of a PV module increases or the electrical characteristics between cells become inhomogeneous, the MPP of the V–P curve shifts toward lower voltage and lower power. Therefore, the peak power value of the curve itself decreases compared to its initial state, and the overall power generation performance deteriorates. Figure 5a and Figure 5b show the I–V/V–P characteristics of shaded and short-circuited PV strings, respectively [19]. In the partially degraded panel section, the slope of the curve changes rapidly, forming inflection points, and the number of those points shows a close correlation with the number of degradation panels.

### 2.5. Existing Methods for Detecting Degradation and Their Limitations

Traditional I–V characteristic curve analysis quantifies degradation rates by monitoring changes in short-circuit current, open-circuit voltage, and MPP using I–V curves measured outdoors. However, this method requires calibration to account for irradiance and temperature at the time of measurement, and power generation is temporarily interrupted during I–V curve recording, thus limiting real-time automated monitoring [20]. AI models utilizing power generation data typically predict module degradation based on deep learning or machine learning techniques applied to power generation and weather data collected over an extended period. However, these methods are highly sensitive to data accuracy and distortion, making it difficult to clearly distinguish and interpret degradation phenomena from external environmental influences such as solar radiation and temperature [21]. Therefore, this study proposes a degradation detection technique that enables real-time automation and cause specific, quantitative diagnosis using the first derivative of the V–P curve and AI models.

## 3. V–P Curve Detection Method Based on Capacitor Power Charging Circuit

In this study, a simple circuit consisting of an SCR and a capacitor power charging circuit connected in series with a solar string in a distribution panel constructed in a solar power system was applied to acquire the V–P curve. Each PV string is individually controlled by a SCR switch, whose self-commutation characteristics stably suppress transient currents. The output of the selected string is applied to the capacitor power charging circuit, and the capacitor voltage $V_{Cs}\left(t\right)$ and $I_{Cs}\left(t\right)$ are measured to extract the V–P curve for each string. In the initial discharge state, the PV string supplies $I_{Cs}$. As $V_{Cs}\left(t\right)$ charges from 0 to $V_{OC}$, the circuit current gradually decreases as described by Equation (2).

(2) $$I_{Cs}\left(t\right)=\frac{V_{OC}-V_{Cs}\left(t\right)}{R_{s}}$$

During this process, by measuring the output voltage of the solar panel and the current flowing through C through the ADC, the V–P curve of the solar panel series string can be obtained through the charging operation of the capacitor. The charging and discharging operations are determined by the time constant of the $R_{s}$–$C_{s}$ circuit, as given by Equation (3).

(3) $$\tau =R_{s}C_{s}$$

In the charging operation, the capacitor voltage $V_{Cs}\left(t\right)$ increases exponentially from the initial value 0 to $V_{OC}$ as shown in Equation (4), and $I_{Cs}$ decays from the initial value $V_{OC}$/$R_{s}$ to 0.

(4) $$\left\{\begin{array}{c} V_{Cs}\left(t\right)=V_{OC}\left(1-e^{-\frac{t}{\tau }}\right)\\ I_{Cs}\left(t\right)=\frac{V_{OC}-V_{Cs}\left(t\right)}{R_{s}}=\frac{V_{OC}}{R_{s}}e^{-\frac{t}{\tau }} \end{array}\right.$$

In contrast, in discharge mode, the capacitor voltage decreases as shown in Equation (5) as it releases the stored charge.

(5) $$\left\{\begin{array}{c} V_{Cs}\left(t\right)=V_{Cs}\left(0\right)e^{-\frac{t}{\tau }}\\ I_{Cs}\left(t\right)=-C_{s}\frac{dV_{Cs}}{dt}=\frac{V_{Cs}\left(0\right)}{R_{s}}e^{-\frac{t}{\tau }} \end{array}\right.$$

The instantaneous power corresponding to the capacitor voltage is defined by Equation (6), which is expressed as the product of the capacitor voltage and the current at the corresponding voltage.

(6) $$P\left(t\right)=V_{Cs}I_{Cs}\left(t\right)$$

**Figure 6:**
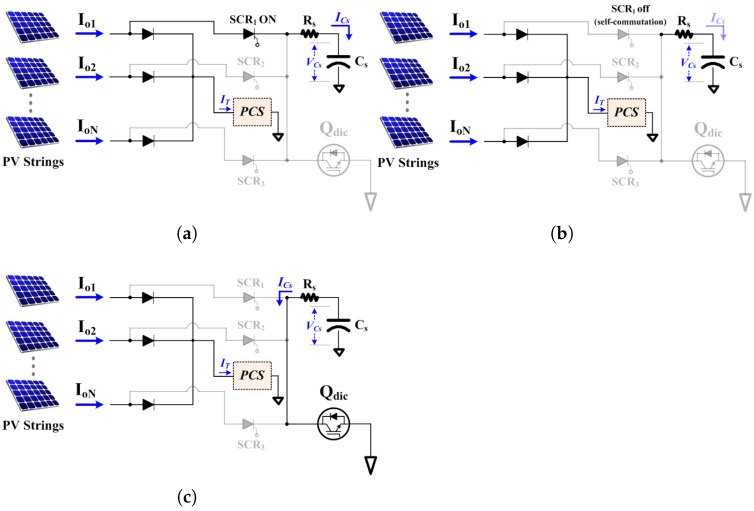
Sequential SCR switching process for individual string diagnosis. (**a**) Only SCR_1_ ON; (**b**) Only SCR_1_ OFF; (**c**) Only $Q_{dic}$ ON.

During the discharge process, the power attenuation curve exhibits distinct attenuation characteristics between the normal and degraded sections. In the normal section, power decreases at a relatively steady and gradual rate. However, in the degraded section, the rapid increase in internal resistance leads to a sharp reduction in current, resulting in an accelerated power attenuation and the formation of a nonlinear region. This rapidly changing power attenuation rate appears as an inflection point in the power curve, which can be effectively used to clearly identify the onset of degradation. The string diagnostic procedure, consisting of SCR switching, self-commutation, and $Q_{dic}$ based discharge is shown step by step in Figure 6. This approach differentiates each string’s behavior based on the exponential decay of the capacitor voltage described in Equation (5), and on the corresponding changes in current.

- Step 1—Figure 6a: Capacitor Charging via SCR Switching
  In the first step, the selected SCR_1_ is turned on, allowing current from the corresponding PV string to flow into the capacitor power charging circuit, charging the capacitor $C_{s}$. The voltage level and charging rate of the capacitor are determined by the output characteristics of the PV string.
- Step 2—Figure 6b: SCR Self-Commutation and Standby Mode
  Once $C_{s}$ is fully charged to the string voltage, the current through the SCR decreases, causing the SCR to naturally turn off through self-commutation. At this point, $C_{s}$ maintains the stored voltage level, entering a standby mode for the subsequent discharge step.
- Step 3—Figure 6c: Direct Capacitor Discharge via Qdic Switching
  In the third step, the $Q_{dic}$ switch is activated by a PWM signal, discharging the stored voltage from $C_{s}$ directly through the $Q_{dic}$ switch. During discharge, the capacitor voltage and current exponentially decrease over time, and the measured voltage and current curves exhibit distinct attenuation characteristics depending on the number of degraded panels within the string.

**Figure 7:**
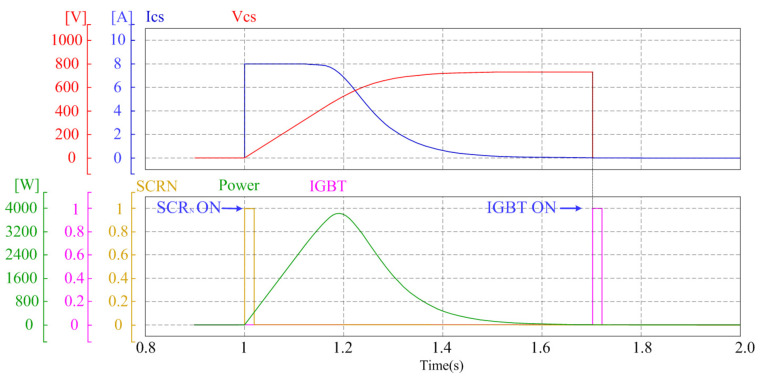
Timing diagram of the sequential SCR switching process.

Figure 7 is a timing diagram that illustrates the previously described three-step string diagnosis process over time. Each signal is directly correlated with the previously discussed circuit operations and mathematical equations. The rate of these charging and discharging operations is governed by the time constant (τ) of the $R_{s}$–$C_{s}$ circuit, which is defined in Equation (3). First, when the SCR ON signal is applied at t = 1.0 s, the capacitor begins to charge. During this process, $I_{Cs}$ exponentially decreases from the initial short-circuit current, while ($V_{Cs}$) gradually increases from 0 to $V_{OC}$, following the behavior described in Equation (4). The instantaneous power, expressed as the product of capacitor voltage and current, creates the V–P curve for diagnosis and is defined by Equation (6). When ($I_{Cs}$) drops near zero, the SCR self-commutates (turns off), and the circuit enters a standby state, holding $V_{Cs}$ constant. Then, at t = 1.7 s, the insulated gate bipolar transistor (IGBT) ON signal triggers the $Q_{dic}$ switch, causing the charge stored in $V_{Cs}$ to start discharging, a process governed by Equation (5). Through this sequential charging and discharging process, the power curve representing the V–P characteristics of each string is successfully extracted, enabling the analysis of the degradation state.

These patterns are utilized as raw data and serve as input features for AI diagnostic models to differentiate between normal conditions, degradation in a single string, and degradation in two or more strings. Three-step diagnostic procedure is sequentially repeated for each string, and the process continues iteratively to enable real-time monitoring. Therefore, unlike I–V curve measurement, the capacitor power charging circuit-based V–P curve diagnostic method is not sensitive to variations in solar irradiance and temperature, as it is based on the analysis of the attenuation pattern, slope, and inflection point locations of the charge and discharge curves for each string. Additionally, by applying string selection using an SCR switch and adopting a sequential measurement approach, power generation does not need to be temporarily suspended, and real-time measurement is achievable even under low-load conditions, resulting in minimal power loss.

In the following section, a method for implementing an LSTM-based AI model is proposed, which automatically predicts the number of degraded panels and the degree of degradation in each string by utilizing the raw data and first-order derivative features of the V–P curve as input variables. The AI model was trained using MATLAB simulations, and the proposed prediction method was validated through actual system operation experiments.

## 4. Design and Implementation of an AI-Based Degradation Diagnosis Model

### 4.1. Data Grouping for Normal/Abnormal State Classification

The method proposed in this study is designed to efficiently diagnose solar panel degradation by utilizing only the electrical characteristics of PV strings, without the need for complex measurement equipment, through the use of SCR and capacitor power charge circuits. To this end, we continuously measured PV string voltage and current by exploiting the SCR’s self-commutation and the charging and discharging dynamics of the capacitor power charging circuit; we then reconstructed the V–P curve from the resulting data. Measurements taken under various single-panel and double-panel degradation conditions enabled a detailed analysis of changes in the power derivative (dP/dV) and the locations of inflection points within the V–P curve.

The analyzed raw data were obtained through simulations using MATLAB, and a dataset was constructed to distinguish between normal and abnormal conditions clearly. In the first step of the proposed degradation detection method, the classification criterion was defined to distinguish between normal and abnormal conditions based on the irradiance value λ. If the irradiance λ is uniformly applied to all PV modules, the state is defined as normal; otherwise, it is classified as abnormal. The maximum solar irradiance (λ) was set to 1000 W/m^2^, in accordance with the Standard Test Conditions, which is the performance measurement standard for the MSX-60 panel used in this study. The resulting dataset (1,099,900 data points) was used as input features: voltage ($V_{Cs}$), power ($P_{N}$), and current ($I_{Cs}$) in columns 1 to 3, the fourth column serving as a binary label.

**Figure 8:**
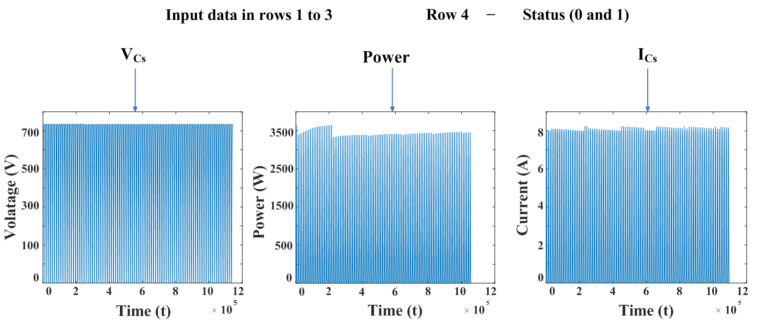
Input features and labeling for PV degradation detection.

Figure 8 shows the input features ($V_{Cs}$, $P_{N}$, $I_{Cs}$) of the raw data in separate graphs, illustrating how each feature value changes over time. Additionally, the entire data was divided into 70% as a training set and 30% as a validation set, and used for training and validation of a shallow network model based on LSTM.

**Figure 9:**
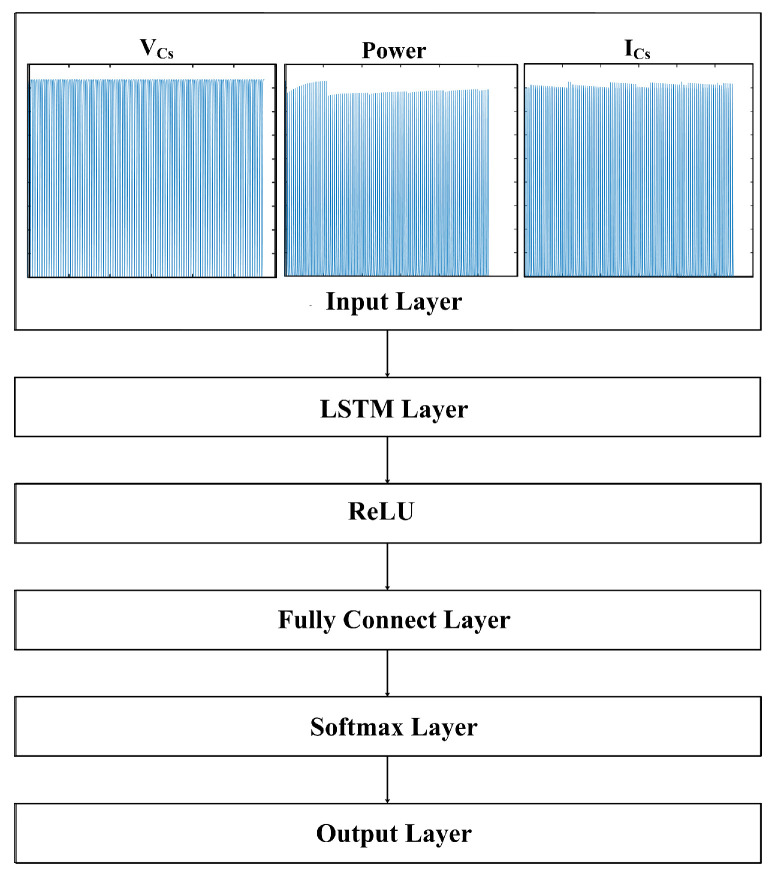
LSTM-based binary classification model architecture.

Figure 9 shows the structure of the LSTM-based binary classification model proposed in this study. The input layer receives the time-series data of power, $V_{Cs}$, and $I_{Cs}$ presented in Figure 8. The LSTM layer sequentially learns power data that changes over time and effectively captures time-series characteristics such as inflection points and waveform distortions caused by degradation. Additionally, by applying the ReLU activation function to introduce nonlinearity, the model is configured to flexibly represent various types of input signals [22]. The fully connected layer integrates the time-series features extracted from the LSTM layer and transforms them into a form suitable for class classification. The softmax layer outputs the probability distribution for each class (normal or abnormal). Finally, the output layer selects the class with the highest probability and classifies the input as either normal (0) or abnormal (1).

This LSTM-based binary classification model is designed by considering the temporal characteristics of MPP curves, where inflection points occur due to the degradation of specific PV modules connected in series within a solar power system. The dataset used in this study has a relatively short time-series length, and changes in key features are clearly distinguishable. Therefore, a shallow model is considered more suitable and efficient than a complex deep neural network. Accordingly, we adopt a shallow two-layer LSTM to reduce vanishing-gradient risk and ensure stable training, while retaining enough capacity to model patterns in 6000-dimensional time-series data. With approximately 2.4 million parameters, the model balances representational capacity and overfitting risk. This design keeps the computational load and memory footprint low, enabling the inference speed required for real-time fault detection. The training and validation results for the proposed data grouping and LSTM model design are presented in detail in Section 5.1 and Section 5.2.

### 4.2. Classification of Degradation Stages Based on Classified Abnormal Data

**Figure 10:**
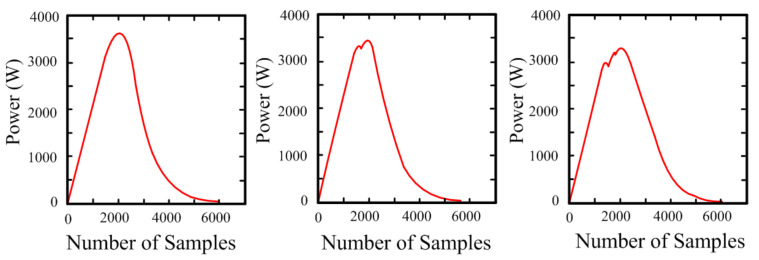
Power waveform under different numbers of degraded modules.

In the final stage of the proposed degradation detection method, which involves classifying specific degradation states, the raw power data corresponding to the previously classified abnormal samples were utilized. To improve classification accuracy, the proposed data preprocessing procedure was applied prior to degradation state classification. Figure 10 shows how the power waveform changes over time according to the number of degraded panels in the string. The three graphs respectively present the time responses of the measured power under normal conditions, one-panel degradation, and two-panel degradation. Under normal conditions, the power output exhibits an ideal pattern, reaching its maximum value after a certain period and then gradually decreasing. By contrast, when one or more degraded panels are present, the power decreases and the P–V curve exhibits increasing distortion around MPP. In particular, as the number of degraded panels increases, the asymmetry of the waveform becomes more pronounced, providing visual information that can be effectively utilized to accurately classify the degradation status within the model.

**Figure 11:**
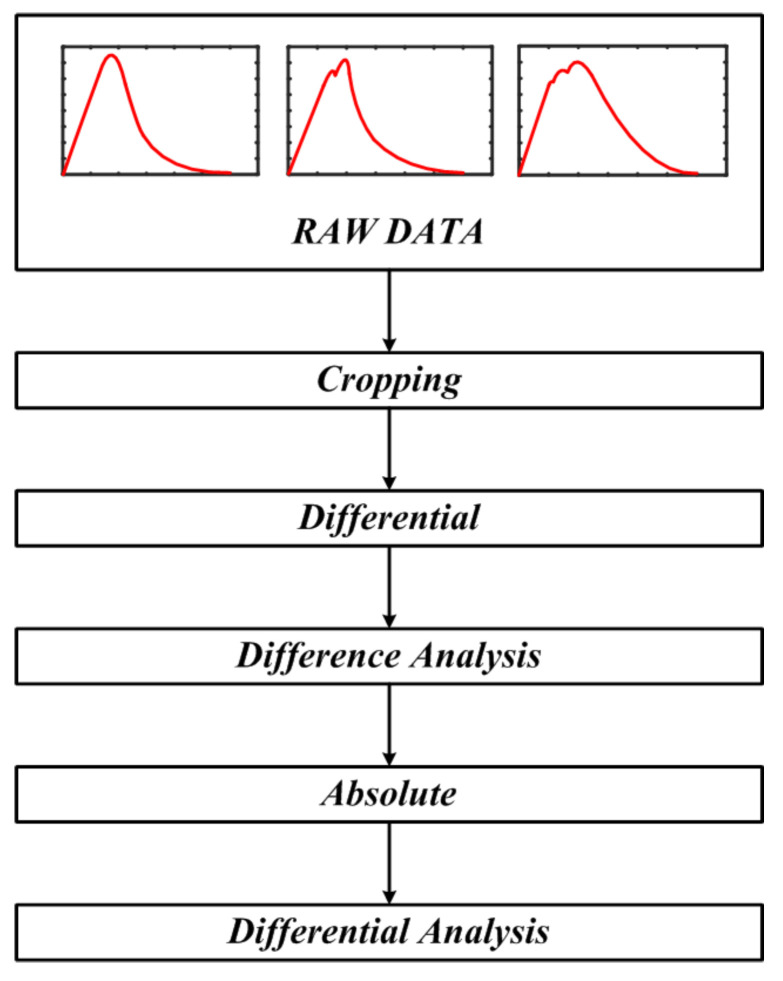
Raw data preprocessing flow for dataset construction.

Based on this raw data, data preprocessing and dataset construction were performed, and the overall process is detailed in Figure 11. The first step, labeled "Raw Data" in Figure 11, corresponds to the power data over time presented in Figure 10. Subsequently, data cropping was applied to select only the time intervals of interest for analysis from the entire collected dataset. This step is essential for removing unnecessary data segments and noise, thereby improving accuracy and optimizing computational efficiency.

We analyzed the 1.0–1.6 s interval (0.6 s; ≈6000 samples) to exclude the initial transient and focus on the steady-state signal. The start time was set to 1.0 s to exclude the initial transient, ensuring that only steady-state data were analyzed. Because a 0.6 s interval yielded sufficient samples, the end time was set to 1.6 s. This setting reflects a comprehensive assessment of (i) the system’s time to steady state derived from response analysis, (ii) comparative performance under alternative windows (0.5 s, 1.0 s, 1.5 s), and (iii) real-time detection constraints. The results indicate that the [1.0–1.5] s interval (0.5 s; ≈5000 samples) offers excellent real-time performance, while the [1.0–1.6] s interval (0.6 s; ≈6000 samples) provides the most favorable accuracy–latency compromise. The [1.0–1.8] s and [0.8–1.6] s windows (0.8 s; ≈8000 samples; ≈0.8 s decision latency) offered greater statistical stability but at the cost of higher latency. From an operational standpoint, we therefore recommend the [1.0–1.6] s interval.

Next, differential operations were applied to extract the rate of change, enabling the sensitive detection of subtle characteristic changes associated with the degradation process. This study employs a derivative to sensitively capture the dynamic characteristics of the system, an approach well-suited for promptly revealing the subtle pattern changes that accompany the progression of degradation. We employed MATLAB’s gradient function to obtain noise-robust numerical derivatives. In particular, the power parameter—used as a key indicator in this study—was selected as the differentiation variable because it exhibits the greatest sensitivity to system degradation. Furthermore, noise sensitivity—quantified as standard deviation (SD)—was 0.1418 for forward differencing, 0.1418 for backward differencing, and 0.0701 for central differencing. The Smoothed Gradient reduced the SD to 0.0020. Consequently, given the risk that excessive smoothing can obscure weak early-stage anomalies, the smoothed-gradient approach was relegated to a secondary role. Central differencing is adopted by default owing to its superior accuracy–noise suppression trade-off. We subsequently applied an absolute-value transform so that the magnitude of change, irrespective of sign, serves as the primary indicator. This prevents changes in opposite directions from canceling out. By mapping all deviations to positive magnitudes, it ensures a consistent representation of the data’s characteristics.

Differential analysis was then conducted on the absolute-valued data to clearly distinguish between normal and abnormal conditions. The resulting differentiated data was then used to clearly distinguish between normal and abnormal conditions. Differential analysis is essential for distinguishing normal data from degradation-induced anomalies, making the characteristic differences between the two groups clearly evident. Through this process, the differences in characteristics between the two data groups become more apparent, enabling machine learning models to maintain high accuracy during subsequent training. Finally, all data were standardized within a specified range through reference normalization. This step was performed to minimize bias in the analysis by eliminating differences in data scale, ensuring accurate comparisons and effective pattern recognition.

**Figure 12:**
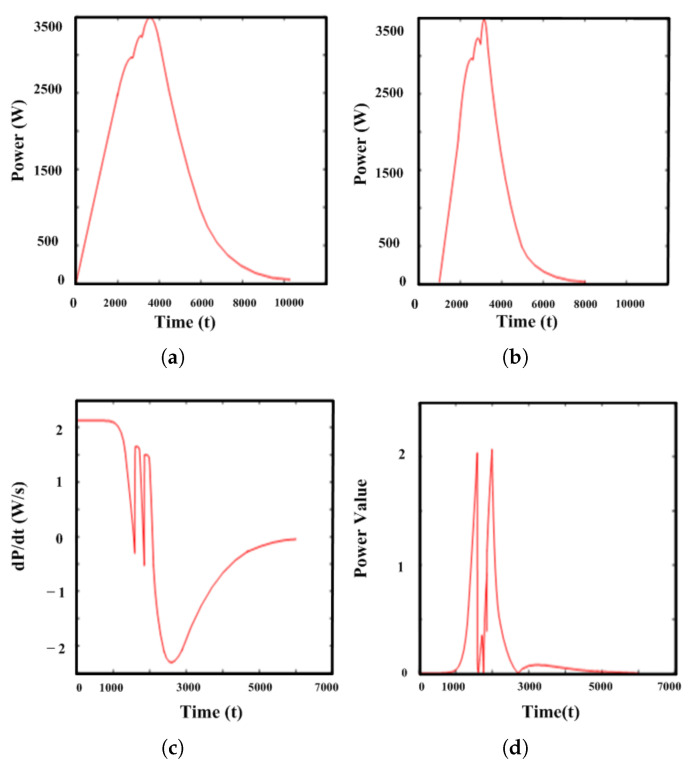
Preprocessing stages for PV degradation dataset construction. (**a**) Raw data; (**b**) Data cropping; (**c**) Differential; (**d**) Difference analysis and absolute-based normalization.

Figure 12 presents the results of each data preprocessing step in graphical form. In Figure 12a, the initial data covers the entire time range and appears relatively long, containing considerable noise and unnecessary information. Since the measured data may contain various types of noise and unnecessary voltage and current fluctuations, it is not suitable to use the raw data directly for quantitative analysis. Therefore, as shown in Figure 12b, signal intervals appropriate for degradation analysis and model training were selectively extracted. As a result, while the overall visual structure was maintained, the preprocessing retained only the essential intervals required for model input, significantly reducing unnecessary noise elements [23,24]. Subsequently, as shown in Figure 12c computes the rate of change to accentuate subtle waveform variations. As degradation progresses, inflection points become more pronounced under differentiation; their positions and shapes capture temporal changes and provide strong cues for separating degraded from normal states. This differential preprocessing better captures degradation than conventional representations. In the final step, shown in Figure 12d, the absolute value of the differentiated data was applied to remove the directionality (positive or negative) of the rate of change and to emphasize the magnitude of the change. Through detailed analysis, normal and abnormal data were clearly distinguished, followed by the application of standard normalization to adjust the scale and range of the data. This process ensured that all data were comparable on a unified scale, significantly improving the efficiency of learning and prediction accuracy of the models. Through this systematic preprocessing procedure, a reliable dataset was constructed to effectively and accurately distinguish between different stages of degradation. The dataset developed in this chapter will be presented in detail in Section 5.2.

## 5. Experiments and Analysis

In this study, the training and evaluation of the artificial intelligence classification model were conducted using MATLAB R2024a in a Windows 11 environment with an NVIDIA RTX 4090 graphics card. The solar module used is the MSX-60 model, as detailed in Table 2, which features a maximum output power of 60 W, $V_{OC}$ of 21.1 V, and $I_{SC}$ of 3.8 A. The dataset was constructed based on raw data obtained through simulation and was labeled as either normal or abnormal for use in the analysis. Based on this dataset, Section 5.1 presents the experimental results of the first stage in the proposed degradation detection framework, focusing on the classification of normal and abnormal conditions. Section 5.2 analyzes the final stage performance for identifying the detailed degradation states of photovoltaic modules.

### 5.1. Results of Experiments Classifying Normal and Abnormal States

In this study, the entire dataset was divided into a 70% training set and a 30% validation set to train and validate the LSTM model for classifying normal and abnormal states. The comparative analysis covered logistic regression, SVM, random forest, a 1D-CNN, and the proposed shallow LSTM. To ensure a fair comparison, all models were evaluated under the same preprocessing pipeline and the same metrics: accuracy, AUC, confusion matrix, and ROC curve. The logistic regression model was constructed according to Equation (7), and the predicted probability *p* was computed using the logistic function.

(7) $$p=\left(\frac{1}{1+\exp\left(-(b_{1}x_{1}+b_{2}x_{2}+b_{3}x_{3})\right)}\right)$$

where *p* represents the probability of belonging to class 1; *x* is the normalized input feature value; $b_{0}$ is the intercept; and $b_{1}$, $b_{2}$, and $b_{3}$ are the regression coefficients for each respective feature. Furthermore, we applied z-score normalization to the SVM to stabilize margin and coefficient estimation. By contrast, the Random Forest being tree-based and largely scale-invariant was trained on the original (unnormalized) features. As a comparative baseline, we used a lightweight 1D-CNN that ingests a 3-D input tensor and stacks two 1-D convolutional blocks (batch normalization + ReLU), global average pooling, a fully connected layer, and a softmax classifier.

**Figure 13:**
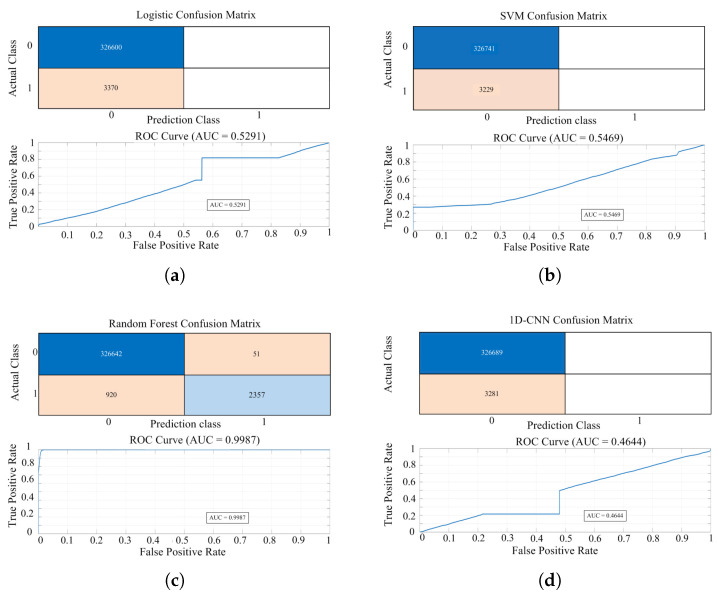
Performance evaluation for normal/abnormal classification of four baseline models: (**a**) Logistic; (**b**) SVM; (**c**) Random Forest; (**d**) 1D-CNN.

Figure 13 reports results for four baselines—(a) logistic regression, (b) SVM, (c) random forest, and (d) 1D-CNN—with each panel showing a confusion matrix and a ROC curve with AUC. Quantitatively, the AUCs for logistic regression (≈0.5291) and SVM (≈0.5469) were comparable, whereas the 1D-CNN produced a lower AUC (≈0.4644). Random forest achieved the highest classification performance (≈0.9987). Its confusion matrix also shows a relatively high recall for the abnormal class (Class 1).

**Figure 14:**
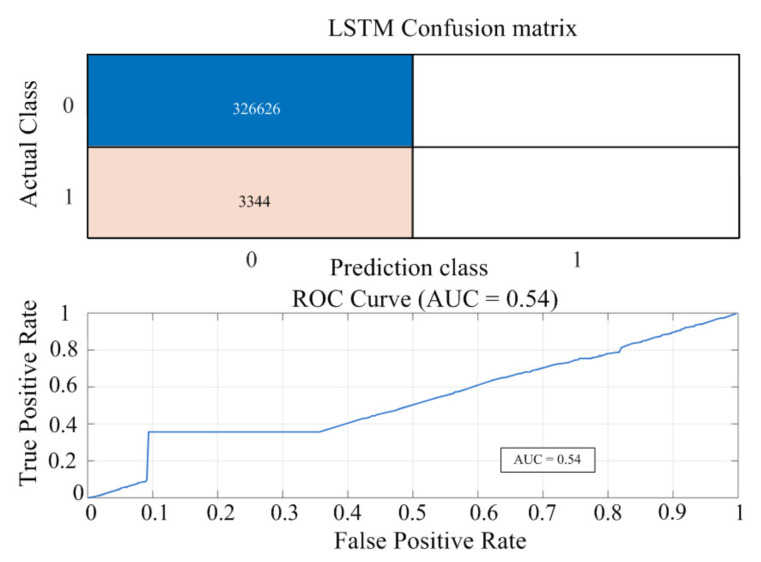
Performance evaluation of the proposed shallow LSTM model (Noramal/Abnormal).

As shown in Figure 14, the proposed shallow LSTM has an AUC of approximately 0.54, which is lower than that of Random Forest but comparable to the performance of Logistic Regression and SVM. This low AUC is a consequence of the severe class imbalance in the dataset (Normal:Abnormal ≈ 99:1). As the AUC metric is highly sensitive to minority class detection, it would be inappropriate to underestimate the model’s performance based solely on this single indicator. The proposed shallow LSTM can capture temporal features, such as inflection points and waveform distortions in the power curve, which is critical for long-term detection of PV module degradation. Accordingly, the results in Figure 14 should be viewed not as an absolute performance comparison, but as evidence that the proposed method can detect abnormal patterns to a meaningful degree even under extreme class imbalance. Random forest achieved a higher AUC, but its memory footprint and inference latency limit real-time deployment. In contrast, the proposed shallow LSTM was selected as the final model for its deployability and scalability to long, multichannel time-series data and amenability to lightweighting, quantization, and hardware acceleration. Table 3 summarizes the training and test accuracies for each model. All models exhibit comparable performance (98–99%), and the small training–validation gap indicates no explicit signs of overfitting.

**Figure 15:**
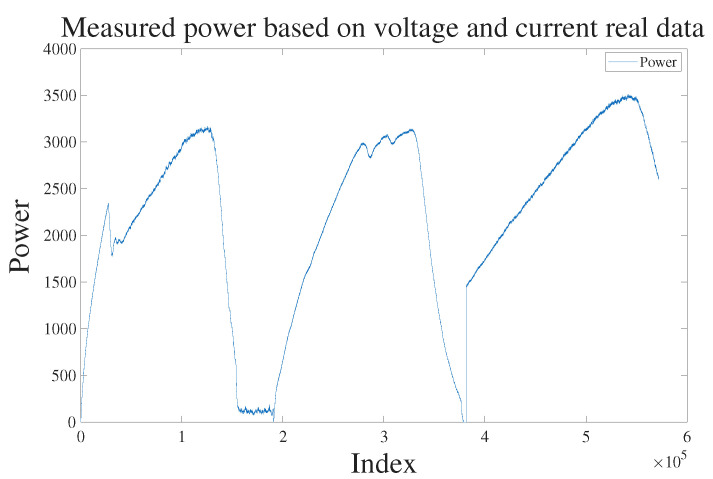
Field-measured power waveforms: inflection points—0 (Normal), 1 (Single-string degradation), 2 (Dual-string degradation).

For the experimental validation, a PV array with uniform characteristics was constructed to simulate artificial degradation, which was induced by partially shading selected modules. The resulting P–V curves displayed a decrease in maximum power and additional inflection points; these features were used as the principal basis for degradation assessment. As shown in Figure 15, the dataset is clearly partitioned into three conditions (Normal, Single-String Damage, Dual-String Damage). Each condition exhibits a distinct power waveform, discernible by its number of inflection points—0, 1, and 2, respectively.

Oscilloscope acquired data were exported to CSV and processed in MATLAB to assess four baseline models and the proposed shallow LSTM. As summarized in Table 4, Random Forest achieved the highest performance (AUC = 1.000; training accuracy = 99.91%; test accuracy = 99.96%). The 1D-CNN was similarly strong (AUC = 0.9993; 97.99%; 97.94%), and the proposed shallow LSTM also yielded competitive results (AUC = 0.9974; 96.41%; 97.07%). By contrast, Logistic Regression (AUC = 0.8676; 78.79%; 78.92%) and SVM (AUC = 0.8702; 77.59%; 77.90%) underperformed. Contrary to the initial simulation-based results, validation with real-world measurements confirmed the effectiveness of the proposed LSTM model. The model appears to capture temporal patterns and waveform characteristics (e.g., inflection points and distortions in the power curve), allowing reliable discrimination of normal vs. abnormal states in noisy field data.

The hyperparameters used for training are presented in Table 5. The model was configured with an input size of 3 and 100 hidden units. The model was trained for 10 epochs with a batch size of 1000. Relative to the 6000-sample sequence length, using 100 hidden units yields ≈ 1:60 compression, preserving salient information while maintaining sufficient capacity. The resulting model size is ≈9.6 MB, supporting low-latency inference under resource constraints. In comparative experiments, 50 units risked insufficient capacity, whereas ≥200 units increased overfitting and computational cost. A hidden size of 100 units provided the best trade-off among performance, model complexity, and inference latency. We set a 100-epoch maximum with early stopping; by stopping before the validation loss rose, we limited overfitting while maintaining adequate training and runtime efficiency. The Adam optimizer was utilized for optimization, and a learning rate of 0.001 was set to ensure stable training. This is a well-established setting for the Adam optimizer; it yields stable, non-oscillatory convergence and enables fine-grained weight updates for precise training [25].

### 5.2. Results of Degradation Stage Classification Experiment

For the training and validation of the model designed to classify degradation stages, the entire dataset was partitioned into a 70% training set and a 30% test set. The comparison was conducted under the same conditions as the normal/abnormal classification, and all models were evaluated using the same preprocessing pipeline and identical metrics (accuracy, AUC, confusion matrix, ROC). We reduced computational complexity by applying PCA with 50 components to the logistic regression model at preprocessing, then computed class probabilities via the logistic function (Equation (8)).

(8) $$p=\frac{1}{1+\exp\left(-(b_{0}+b_{1}x_{1}+b_{2}x_{2}+\ldots +b_{n}x_{n})\right)}$$

where *p* represents the predicted probability of belonging to the positive class (class 1); $x_{1}$, $x_{2}$, …, $x_{n}$ are the n principal components obtained from PCA; is the intercept; and $b_{1}$, $b_{2}$, …, $b_{n}$ are the regression coefficients for each corresponding principal component.

**Figure 16:**
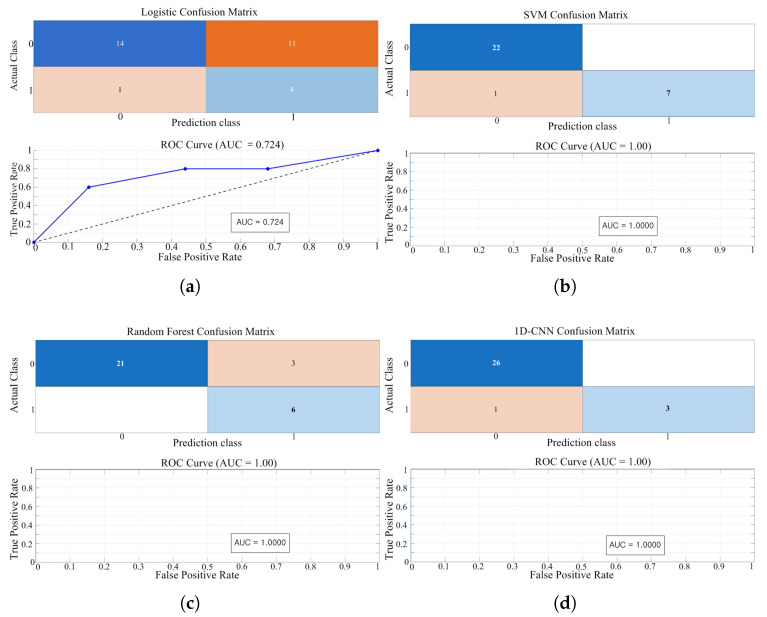
Performance evaluation for degradation-stage classification of four baseline models: (**a**) Logistic; (**b**) SVM; (**c**) Random Forest; (**d**) 1D-CNN.

**Figure 17:**
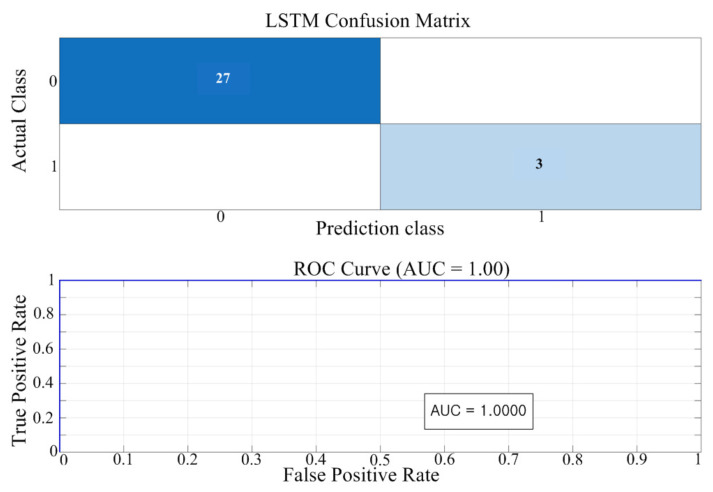
Performance evaluation of the proposed shallow LSTM model (Degradation-stage classification).

We applied z-score normalization to the SVM. By contrast, the random forest—being tree-based and largely scale-invariant—was trained on the original (unnormalized) features. The 1D-CNN baseline employed a lightweight stack of two 1D conv blocks (batch normalization + ReLU), global average pooling, a fully connected layer, and a softmax output. Figure 16 presents the confusion matrices and ROC/AUC curves for the four baseline models—(a) Logistic Regression, (b) SVM, (c) Random Forest, and (d) 1D-CNN—while Figure 17 shows the results for the proposed LSTM model. The quantitative results are as follows. Logistic regression exhibited limited classification performance (AUC = 0.724; training accuracy = 60.00%; test accuracy = 42.03%). In the confusion matrix, 11 of 25 normal (Class 0) samples were misclassified as degraded (false positives; see Figure 16a). The SVM model exhibited high performance, attaining an AUC of 1.00 with training and test accuracies of 100.0% and 96.67%, respectively (Figure 16b). The random forest model also performed well (AUC = 1.00; 97.10%/90.00%), though some misclassifications were present (Figure 16c). The 1D-CNN also performed well, achieving AUC = 1.00 with training/test accuracies of 100.0%/96.67% (Figure 16d). The proposed LSTM achieved AUC = 1.00 and training/test accuracies of 100.0%/100.0%; in the confusion matrix, it correctly classified all 27 normal and 3 degraded samples (Figure 17). This indicates that validation performance remained stable throughout training, suggesting that the model learned the degradation characteristics without overfitting. These results are summarized in Table 6, which reports the AUC and the training/test accuracies for all models.

In summary, logistic regression was insufficient for discriminating between normal and degraded states in this study, whereas SVM, random forest, and 1D-CNN exhibited near-perfect classification. The proposed shallow LSTM showed the most stable behavior across AUC and accuracy, reaching AUC 1.00 and 100.0% accuracy and classifying all samples correctly.

The key hyperparameters used for training are listed in Table 7. An input size of 6000 and 100 hidden units were set for the model. Similar to the methodology in Section 5.1, the length of the input sequence included only the data essential for the training process. Similar to the previous model, the number of hidden units was varied across tests (10, 50, 100, 150, and 200). It was determined that the analysis performed most stably with the hidden unit count set to 100. The model was trained for 100 epochs with a batch size of 1000. The Adam optimizer was utilized for optimization, and a learning rate of 0.001 was set to ensure stable training.

**Figure 18:**
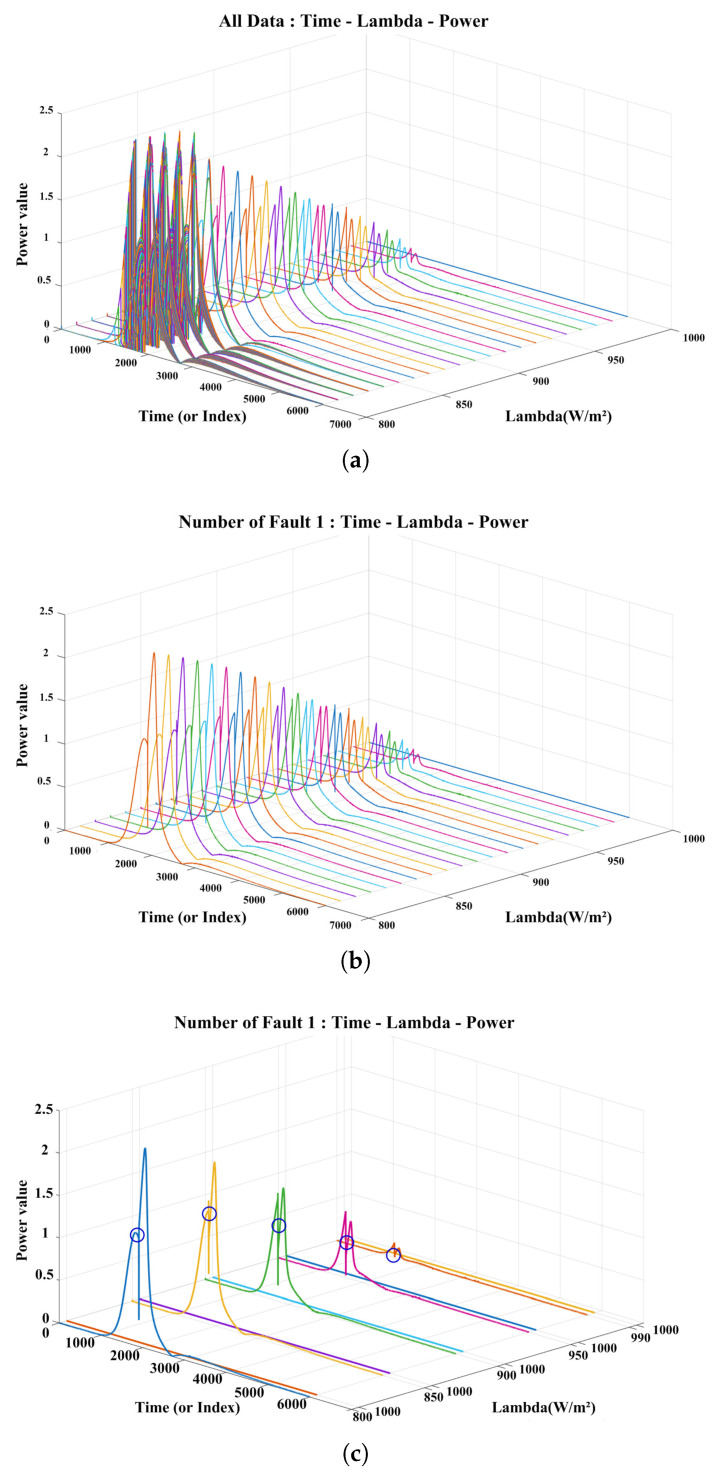

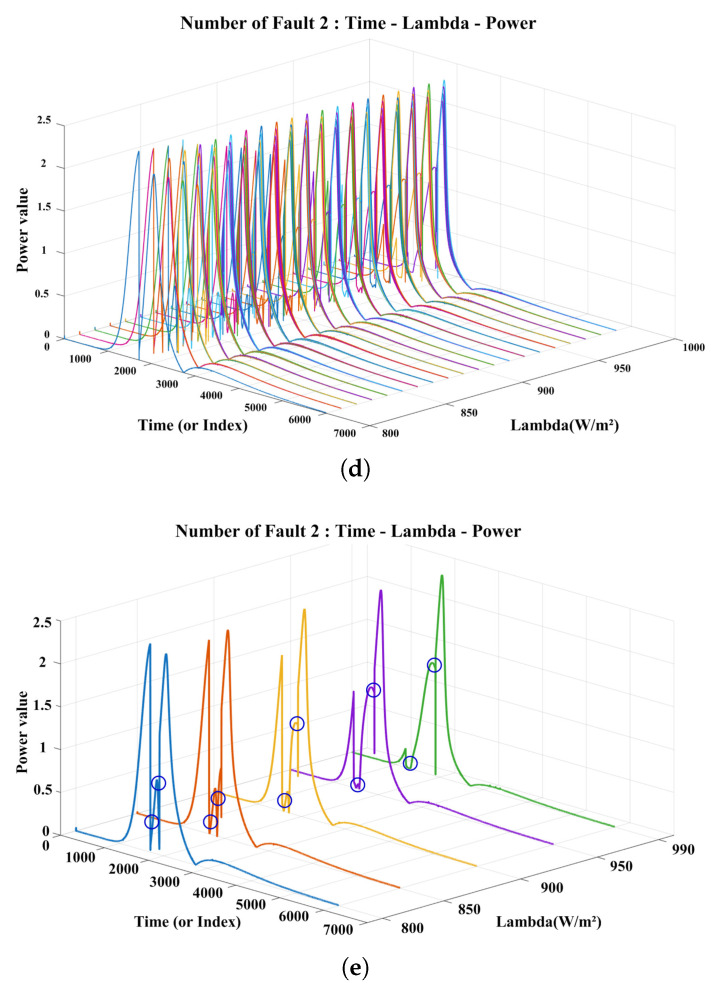
3D Visualization of time–lambda–power surface according to fault condition. (**a**) all data; (**b**) one degraded string; (**c**) comparison at selected λ values under (**b**) (inflection points marked); (**d**) two degraded strings; (**e**) comparison at selected λ values under (**d**) (inflection points marked).

Figure 18 shows the differences in output characteristics across various degradation states by visualizing the normalized data in three dimensions. Figure 18a shows the visualization results for the entire dataset, with time, lambda, and power as the axes, providing a visual representation of how the normal and degraded states are distributed across the full lambda spectrum. A noticeable pattern of decreasing output along the time axis is observed, which can be interpreted as the effect of degradation factors such as increased resistance or current loss within the string. Figure 18b shows the power waveform when a single panel is degraded. All curves exhibit similar inflection points; however, the output amplitude gradually decreases as the lambda value increases. Although the basic shape of the curve remains consistent, differences in the distribution of the output amplitude indicate that the degraded panel influences the overall system output. The inflection point’s amplitude, position, and slope change systematically with the degree of degradation. These attributes serve as sensitive diagnostic indicators of the degradation state. Figure 18c compares curves extracted from Figure 17b at irradiance levels of 800, 850, 900, 950, 990, and 1000 W/m^2^, using 1000 W/m^2^ as the baseline. For ease of interpretation, each curve is annotated with an inflection-point marker indicating where the curvature changes. By construction of the normalization, the curve for λ = 1000 W/m^2^ appears as a flat baseline with no inflection, whereas curves with λ < 1000 W/m^2^ exhibit distinct peaks and inflection points. The peak deviation from the baseline is maximal at λ = 800 W/m^2^; as λ increases from 800 to 990 W/m^2^, both the peak height and the inflection-point amplitude gradually diminish, converging toward the baseline. Figure 18d shows the condition in which two panels are degraded. In certain sections, abnormal sharp drops in output or irregular waveform distortions are observed, visually confirming that multiple abnormal conditions cumulatively affect the overall system response. Figure 18e shows a detailed analysis of the fault condition in Figure 18d by extracting key lambda values (800, 850, 900, 950, 990 W/m^2^). In contrast to the single-panel degradation case Figure 18c, each λ curve exhibits two distinct peaks, a signature feature that emerges when two panels are degraded. Markers were placed on these peaks to enable easy identification of changes in the positions and magnitudes of key feature points within the complex waveform.

These visualization results enable a clear comparison of how output waveforms vary under different degradation conditions and provide a basis for the quantitative analysis of key characteristics, such as the occurrence and number of inflection points and changes in curve slope. Based on these findings, a solid foundation was established for effectively distinguishing between single and multiple anomaly conditions, and the validity of the proposed degradation stage classification model was experimentally verified.

## 6. Conclusions

This study proposes a new methodology for accurately and quickly diagnosing module degradation, a representative problem in solar power generation systems. To this end, the output voltage and current characteristics of each PV string were measured using a simple capacitor power charging circuit. The output of the selected string was applied to a capacitor power charging circuit, and the capacitor voltage and circuit current were measured in real-time to extract the V–P curve for each string. It was experimentally demonstrated that the V–P curve characteristics of normal panels, one degraded panel, and two degraded panels could be effectively distinguished by applying the SCR switching technique. The proposed shallow LSTM was initially developed and validated under an inflection-point–based diagnostic framework using simulation data; the approach was subsequently extended to field measurements acquired by an oscilloscope. Despite a relatively low AUC in the severely imbalanced simulation setting, validation on oscilloscope-measured data produced a high AUC, thereby confirming the method’s effectiveness. In future work, we will expand the scale of field data collection and the observation period to further improve the model’s generalization and diagnostic accuracy. The proposed shallow LSTM was ultimately chosen for on-site deployment owing to its scalability to long, multichannel time-series data and its compatibility with model-compression techniques, including quantization and hardware acceleration. For practical IoT/embedded applications, we plan to implement int8 quantization alongside class-imbalance corrections such as class weighting and threshold tuning. Additionally, we will establish an operational pipeline that employs autoencoders and a one-class SVM as supplementary approaches when abnormal data are scarce. The method proposed in this study is designed to deliver both high accuracy and real-time performance for the early diagnosis of photovoltaic (PV) module degradation. Accordingly, it can provide a crucial foundation for the long-term stability of renewable-energy systems and support progress toward sustainable development. 

## Figures and Tables

**Table 1:** Stress factors and influence of solar modules.

No.	Failure Mode	Stress Factor	Module Impact
1	Microcracks	Mechanical stress, thermal cycling	Increased $R_{s}$, partial shading effect
2	PID	High voltage, high humidity	Reduction in $V_{OC}$ and $I_{Cs}$, FF decrease, efficiency loss
3	Hot-Spot	Cell mismatch, shading, cracks	Cell damage, I–V curve distortion, MPP voltage drop
4	LID	Initial light exposure, boron-oxygen complex activation	Reduction in $V_{OC}$ and $I_{Cs}$, efficiency loss
5	LeTID	Prolonged light soaking at elevated temperature	Progressive power loss

**Table 2:** Specifications and parameters of the simulation circuit.

Note	Symbol	Value
Maximum power	$P_{max}$	60 W
Open-circuit voltage	$V_{OC}$	21.1 V
Short-circuit current	$I_{SC}$	3.8 A
Voltage $P_{max}$	$V_{mp}$	17.1 V
Current $P_{max}$	$I_{mp}$	3.5 A

**Table 3:** Performance on normal/abnormal classification: four baselines and the proposed shallow LSTM (AUC, training/test accuracy).

Model	AUC	Training Accuracy [%]	Test Accuracy [%]
Logistic Regression	0.5291	99.01	98.98
SVM	0.5469	98.99	99.02
Random Forest	0.9987	99.87	99.70
1D-CNN	0.4464	99.00	99.01
LSTM	0.5400	99.01	98.98

**Table 4:** Performance on real-data normal/abnormal classfication: four baseline and the proposed shallow LSTM (AUC, training/test accuracy).

Model	AUC	Training Accuracy [%]	Test Accuracy [%]
Logistic Regression	0.8676	78.79	78.92
SVM	0.8702	77.59	77.90
Random Forest	1.0000	99.91	99.96
1D-CNN	0.9993	97.99	97.94
LSTM	0.9974	96.41	97.07

**Table 5:** Normal/abnormal classification model hyperparameters.

Parameters	Parameters Values
Input Size	3
Number of Hidden Units	100
Number of Epochs	10
Batch Size	1000
Adam optimizer Learning rate	0.001

**Table 6:** Performance on degradation-stage classification: four baselines and the proposed shallow LSTM (AUC, training/test accuracy).

Model	AUC	Training Accuracy [%]	Test Accuracy [%]
Logistic Regression	0.724	60.0	42.03
SVM	1.0	100.0	96.67
Random Forest	1.0	97.10	90.00
1D-CNN	1.0	100.0	96.67
LSTM	1.0	100.0	100.0

**Table 7:** Degradation stage classification hyperparameters.

Parameters	Parameters Values
Input Size	6000
Number of Hidden Units	100
Number of Epochs	100
Batch Size	1000
Adam optimizer Learning rate	0.001

## Data Availability

The datasets used and/or analysed during the current study available from the corresponding author on reasonable request.

## References

[1] Alimi O.A., Meyer E.L., Olayiwola O.I. (2022). Solar Photovoltaic Modules’ Performance Reliability and Degradation Analysis—A Review. Energies.

[2] Atia D.M., Hassan A.A., El-Madany H.T., Eliwa A.Y., Zahran M.B. (2022). Degradation and Energy Performance Evaluation of Mono-Crystalline Photovoltaic Modules in Egypt. Sci. Rep..

[3] Di Lorenzo G., Araneo R., Mitolo M., Niccolai A., Grimaccia F. (2020). Review of O&M practices in PV plants: Failures, solutions, remote control, and monitoring tools. IEEE J. Photovolt..

[4] Daxini R., Anderson K.S., Stein J.S., Theristis M. (2025). Photovoltaic Module Spectral Mismatch Losses Due to Cell-Level EQE Variation. IEEE J. Photovolt..

[5] Hopwood M.W., Stein J.S., Braid J.L., Seigneur H.P. (2022). Physics-based method for generating fully synthetic iv curve training datasets for machine learning classification of pv failures. Energies.

[6] Sun Y., Lu L., Wu F., Xiao S., Sha J., Zhang L. (2023). Error Analysis of a Coordinate Measuring Machine with a 6-DOF Industrial Robot Holding the Probe. Actuators.

[7] Li F., Colvin D.J., Buddha V.S.P., Davis K.O., Tamizhmani G. (2024). Electroluminescence and infrared imaging of fielded photovoltaic modules: A complementary analysis of series resistance-related defects. Sol. Energy.

[8] Islam M., Rashel M.R., Ahmed M.T., Islam A.K.M.K., Tlemçani M. (2023). Artificial intelligence in photovoltaic fault identification and diagnosis: A systematic review. Energies.

[9] Elsaraiti M., Merabet A. (2022). Solar Power Forecasting Using Deep Learning Techniques. IEEE Access.

[10] Hu J., Lim B.H., Tian X., Wang K., Xu D., Zhang F., Zhang Y. (2024). A comprehensive review of artificial intelligence applications in the photovoltaic systems. CAAI AIR.

[11] Badr M.M., Hamad M.S., Abdel-Khalik A.S., Hamdy R.A., Ahmed S., Hamdan E. (2021). Fault identification of photovoltaic array based on machine learning classifiers. IEEE Access.

[12] Lee S., Bae S., Park S.J., Gwak J., Yun J., Kang Y., Lee H.S. (2021). Characterization of Potential-Induced Degradation and Leakage Currents in CIGS Solar Cells. Energies.

[13] da Silva M.K., Gul M.S., Chaudhry H. (2021). Review on the sources of power loss in monofacial and bifacial photovoltaic technologies. Energies.

[14] Mahmood F.I., TamizhMani G. Impact of Anti-soiling Coating on Potential Induced Degradation of Silicon PV modules. Proceedings of the 2022 IEEE 49th Photovoltaics Specialists Conference (PVSC).

[15] Dhimish M., Badran G. (2021). Recovery of Photovoltaic Potential-Induced Degradation Utilizing Automatic Indirect Voltage Source. IEEE Trans. Instrum. Meas..

[16] Kim K.A., Krein P.T. Photovoltaic hot spot analysis for cells with various reverse-bias characteristics through electrical and thermal simulation. Proceedings of the IEEE Workshop on Control and Modeling for Power Electronics (COMPEL).

[17] Dhimish M., Mather P., Holmes V. (2019). Novel photovoltaic hot-spotting fault detection algorithm. IEEE Trans. Device Mater. Reliab..

[18] Nam W., Choi J., Kim G., Hyun J., Ahn H., Park N. (2025). Predicting Photovoltaic Module Lifespan Based on Degradation Modes under Outdoor Environments. Energies.

[19] Vargas J.P., Goss B., Gottschalg R. (2015). Large scale PV systems under non-uniform and fault conditions. Sol. Energy.

[20] Li B., Diallo D., Migan-Dubois A., Delpha C. (2023). Performance evaluation of IEC 60891:2021 procedures for correcting I–V curves of photovoltaic modules under healthy and faulty conditions. Prog. Photovolt. Res. Appl..

[21] Massaoudi M., Chihi I., Abu-Rub H., Refaat S.S., Oueslati F.S. (2021). Convergence of Photovoltaic Power Forecasting and Deep Learning: State-of-Art Review. IEEE Access.

[22] Li B., Delpha C., Migan-Dubois A., Diallo D. (2021). Fault diagnosis of photovoltaic panels using full I–V characteristics and machine learning techniques. Energy Convers. Manag..

[23] Wu Y., Chen Z., Wu L., Lin P., Cheng S., Lu P. (2017). An intelligent fault diagnosis approach for PV array based on SA-RBF kernel extreme learning machine. Energy Procedia.

[24] Mansouri M., Trabelsi M., Nounou H., Nounou M. (2021). Deep Learning-Based Fault Diagnosis of Photovoltaic Systems: A Comprehensive Review and Enhancement Prospects. IEEE Access.

[25] Shao Y., Wang J., Sun H., Yu H., Xing L., Zhao Q., Zhang L. (2024). An Improved BGE-Adam Optimization Algorithm Based on Entropy Weighting and Adaptive Gradient Strategy. Symmetry.

